# Factors Related with CH_4_ and N_2_O Emissions from a Paddy Field: Clues for Management implications

**DOI:** 10.1371/journal.pone.0169254

**Published:** 2017-01-12

**Authors:** Chun Wang, Derrick Y. F. Lai, Jordi Sardans, Weiqi Wang, Congsheng Zeng, Josep Peñuelas

**Affiliations:** 1 Institute of Geography, Fujian Normal University, Fuzhou, China; 2 Key Laboratory of Humid Subtropical Eco-geographical Process, Ministry of Education, Fujian Normal University, Fuzhou, China; 3 Department of Geography and Resource Management, The Chinese University of Hong Kong, Hong Kong SAR, China; 4 CSIC, Global Ecology Unit CREAF-CEAB-CSIC-UAB. 08913 Cerdanyola del Vallès. Catalonia. Spain; 5 CREAF. 08913 Cerdanyola del Vallès. Catalonia. Spain; Tennessee State University, UNITED STATES

## Abstract

Paddy fields are major sources of global atmospheric greenhouse gases, including methane (CH_4_) and nitrous oxide (N_2_O). The different phases previous to emission (production, transport, diffusion, dissolution in pore water and ebullition) despite well-established have rarely been measured in field conditions. We examined them and their relationships with temperature, soil traits and plant biomass in a paddy field in Fujian, southeastern China. CH_4_ emission was positively correlated with CH_4_ production, plant-mediated transport, ebullition, diffusion, and concentration of dissolved CH_4_ in porewater and negatively correlated with sulfate concentration, suggesting the potential use of sulfate fertilizers to mitigate CH_4_ release. Air temperature and humidity, plant stem biomass, and concentrations of soil sulfate, available N, and DOC together accounted for 92% of the variance in CH_4_ emission, and Eh, pH, and the concentrations of available N and Fe^3+^, leaf biomass, and air temperature 95% of the N_2_O emission. Given the positive correlations between CH_4_ emission and DOC content and plant biomass, reduce the addition of a carbon substrate such as straw and the development of smaller but higher yielding rice genotypes could be viable options for reducing the release of greenhouse gases from paddy fields to the atmosphere.

## Introduction

Climate change is a major environmental problem of the 21^st^ century caused mainly by increasing emissions of anthropogenic greenhouse gases (GHGs). Agriculture contributes about 20% of the present atmospheric GHG concentration[1]. Methane (CH_4_) and nitrous oxide (N_2_O) are the two most important GHGs from agriculture, with global-warming potentials (GWP) of 28 and 265 CO_2_-equivalents, respectively, on a 100-year time horizon [2]. The atmospheric concentrations of CH_4_ and N_2_O have increased rapidly from preindustrial levels of 722 and 270 ppb to present levels of 1830 and 324 ppb, respectively[2]. N_2_O is also the dominant gas that is catalytically destroying the stratospheric ozone layer, which is harmful to human health [3]. Reducing GHG emissions to the atmosphere is urgently needed to mitigate the adverse impacts of climate change.

CH_4_ emissions from biogenic sources account for more than 70% of the global CH_4_ emissions[4]. Paddy fields are major man-made sources of CH_4_ emissions, accounting for 5–19% of the global anthropogenic CH_4_ budget[5]. Rice is the major cereal crop for more than half of the world’s population[6], and the FAO[7] has estimated that rice production needs to be increased by 40% by the end of 2030s to meet the rising demand from the ever-increasing population. This increased production may lead to increased emissions of CH_4_[8] and may require a higher application of nitrogenous fertilizers to paddy fields, which can lead to increased emissions of N_2_O to the atmosphere[9].

The total CH_4_ and N_2_O emissions from paddy fields mainly depend on a number of microbial-mediated processes in soils, e.g. CH_4_ production, CH_4_ oxidation, nitrification, and denitrification, and on numerous pathways of gas transport, e.g. plant-mediated transport (through the aerenchyma), molecular diffusion, and ebullition[10]. CH_4_ is produced in anaerobic zones by methanogens, 60–90% of which is subsequently oxidized by methanotrophs in the aerobic zones of the rhizosphere and converted to CO_2_[11]. N_2_O is a by-product of nitrification and denitrification. These processes are influenced by many environmental factors such as atmospheric, plant, and soil properties [12–14]. In general, the process-based understanding for CH_4_ and N_2_O have been well-developed whereas field measurements are lacking [11, 15–17]. The availability of electron acceptors and donors in soils plays a key role in regulating CH_4_ and N_2_O production and consumption[18]. Electron acceptors (e.g. Fe^3+^, NO_3_^-^, and sulfate) are reduced during wet periods but regenerated (oxidized) during dry periods[19]. Soils can also provide carbon substrates to microbes for mediating CH_4_ and N_2_O production and enhancing plant growth that in turn governs more than 90% of CH_4_ transport [11]. Plant characteristics (e.g. biomass and root exudation) are also important regulators of CH_4_ and N_2_O metabolism in soils [20]. Other environmental variables, including soil temperature, pH, redox potential (Eh), and soil salinity also influence CH_4_ and N_2_O metabolism [21, 22]. CH_4_ and N_2_O emissions from paddy fields are strongly influenced by environmental factors that vary both spatially and temporally [23]. The individual processes of CH_4_ metabolism and transport and the temporal variability of CH_4_ and N_2_O emissions, which are essential for simulating GHG emissions from paddy fields, however, have rarely been quantified.

China is a major rice-producing country, accounting for 18.7% of the total area of rice paddy fields (3.06 × 10^7^ ha) and 28.6% of rice production (2.06 × 10^8^ Mg) globally [24]. Rice paddy fields contribute 9% of the total agricultural GHG emissions (1.59 × 10^9^ t CO_2_ equivalent) from China [25]. Understanding the dominant processes of CH_4_ and N_2_O exchange and their main controlling factors is important for developing appropriate strategies to mitigate GHG emissions.

We hypothesized that soil and plant properties that can be changed by management in a rice cropland have a significant role in the processes underlying the processes from CH_4_ and N_2_O production, oxidation, and transport until final emission. We then tested this hypothesis by: (1) quantifing the magnitude of GHG (CH_4_ and N_2_O) emissions from a paddy field in Fujian Province in China, (2) examining the temporal variations of production, oxidation, transport, and porewater concentration of CH_4_ and N_2_O in the paddy soil, and (3) investigating the relationships between soil physiochemical properties and CH_4_ and N_2_O metabolism (production, oxidation, transport, and final emission).

The objectives of the present study were to: (1) quantify the magnitude of GHG (CH_4_ and N_2_O) emissions from a paddy field in Fujian Province in China, (2) examine the temporal variations of production, oxidation, transport, and porewater concentration of CH_4_ in the paddy soil, and (3) investigate the relationships between soil physiochemical properties and CH_4_ and N_2_O metabolism (production, oxidation, transport, and final emission).

## Material and Methods

### Study area

**Fig 1:**
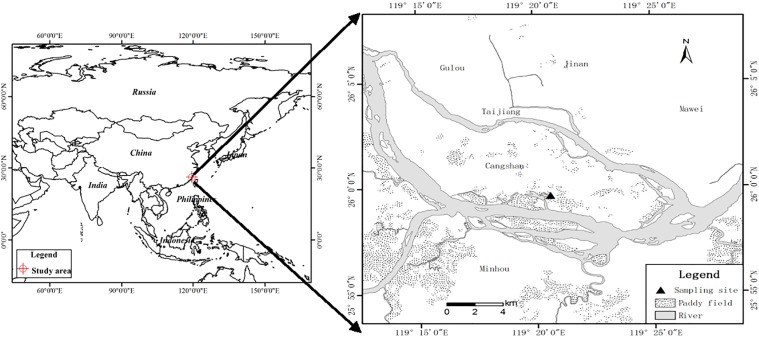
Location of the study area and sampling site (▲) in southeastern China.

The field experiments were carried out at the Wufeng Agronomy Field of the Fujian Academy of Agricultural Sciences (26.1°N, 119.3°E) in southeastern China during the early rice-growing season (April^-^July) in 2011 (Fig 1). The soil of the wetland paddy field was poorly drained, and the proportions of sand, silt, and clay particles in the top 15 cm were 28, 60, and 12%, respectively. Other soil properties (0–15 cm) at the onset of the experiment were: bulk density of 1.1 Mg m^-3^, pH (1:5 with H_2_O) of 6.5, organic-carbon content of 18.1 g kg^-1^, total nitrogen (N) content of 1.2 g kg^-1^, total phosphorus (P) content of 1.1 g kg^-1^, total potassium (K) content of 7.9 g kg^-1^, and available-S of 1.26 mg kg^-1^ After rice transplant, the paddy field was flooded by 5–7 cm of water above the soil surface throughout the growing period (32 days) by using an automatic water-level controller. At the final tiller, water was drained and crop was non-flooded during about one week. Thereafter there was a period with alternating wet and drying treatments and when the rice was ripe, a dry period of about one week was stablished. After this, crop was harvested. The field was plowed to a depth of 15 cm with a moldboard plow and leveled two days before rice transplantation. Mineral fertilizers were applied in three splits as complete (NH_4_-P_2_O_5_-K_2_O, 16-16-16%; Keda Fertilizer Co., Ltd., Jingzhou, China) and urea (46% N) fertilizers. The ratios between N in complete NPK fertilizer and N from urea were 21:1, 7:3 and 9:4 in the first, second and third fertilization times, respectively. Fertilization management in the rice crop followed the typical practices of southern China. A basal fertilizer was applied one day before transplantation at a rate of 42 kg N ha^-1^, 40 kg P_2_O_5_ ha^-1^, and 40 kg K_2_O ha^-1^. Twenty-one-day-old seedlings (three seedlings per hill) of rice (*cv*. Hesheng 10, China) were transplanted manually at a spacing of 14 × 28 cm on 6 April to three replicate plots (10 × 10 m) at the study site. The second split of fertilizer was broadcasted during the tiller initiation stage (seven days after transplanting (DAT)) at a rate of 35 kg N ha^-1^, 20 kg P_2_O_5_ ha^-1^, and 20 kg K_2_O ha^-1^. The third split was broadcasted during the panicle initiation stage (56 DAT) at a rate of 18 kg N ha^-1^, 10 kg P_2_O_5_ ha^-1^, and 10 kg K_2_O ha^-1^. As is habitual in this area we used butachlor at 1.5 kg ha^-1^ two days before rice transplantation to remove small grasses. No specific permissions were required for these locations/activities, the location only have plant rice that no need protect, and our study experiment were also safety. Moreover, the field studies did not involve endangered or protected species.

### Measurement of CH_4_ and N_2_O emissions from the paddy field

Static closed chambers were used to measure CH_4_ and N_2_O emissions during the growing period as described by Datta et al.[23]. The chambers were made of PVC and consisted of two parts: an upper transparent compartment (100 cm height, 30 cm width, 30 cm length) was placed on a permanently installed bottom collar (10 cm height, 30 cm width, 30 cm length). Three replicate chambers were used. Each of these chambers was placed in each plot. Fluxes were measured in triplicate (three days) each week in each plot. Gas samples were collected twice daily. We used the average of the resulting 3x3x2 measurements. Each chamber was installed with two battery-operated fans to homogenize the air inside the chamber headspace, a thermometer to monitor temperature changes during the gas-sampling period, and a gas-sampling port with a neoprene rubber septum at the top of the chamber for collecting gas samples from the headspace. Each chamber covered two rice hills. A wooden boardwalk was built for accessing the plots in the study area to minimize soil disturbance during gas sampling.

Gas fluxes were measured in triplicate weekly at all chamber locations to study the seasonal variation. A 100-ml plastic syringe equipped with a 3-way stopcock was used to collect gas samples from the chamber headspace 0, 15, and 30 min after chamber deployment. Gas samples were collected twice a day. The collected gas samples were immediately transferred to 100-ml air-evacuated aluminum foil bags (Delin Gas Packaging Co., Ltd., Dalian, China) sealed with a butyl rubber septum and transported to the laboratory for analysis of CH_4_ and N_2_O. Additional headspace gas samples were collected hourly from 9:00 to 6:00 the following day to study the diurnal variation of CH_4_ and N_2_O emissions during the tillering (36 DAT) and maturity (85 DAT) stages of the rice crop.

### Determination of CH_4_ and N_2_O concentrations in the headspace air samples

CH_4_ and N_2_O concentrations in the headspace air samples were determined by a gas chromatograph (Shimadzu GC-2014, Kyoto, Japan) packed with a Porapak Q column (2 m length, 4 mm OD, 80/100 mesh, stainless steel column). A flame ionization detector (FID) and an electron capture detector (ECD) were used for the determination of CH_4_ and N_2_O concentrations, respectively. Helium (99.999% purity) was used as a carrier gas (30 ml min^-1^), and a make-up gas (95% argon and 5% CH_4_) was used for the ECD. Calibration was conducted with 1.01, 7.99, and 50.5 μl CH_4_ l^-1^ in He and 0.2, 0.6, and 1.0 μl N_2_O l^-1^ in He (CRM/RM Information Center of China) as primary standards.

### Measurement (in situ) of rates of CH_4_ production and oxidation

The rates of CH_4_ production and oxidation were measured once every two weeks using acetylene (C_2_H_2_) inhibition, which has been successfully used in both laboratory incubations and field-based studies [26–29]. After gas sampling for determining overall CH_4_ emission, C_2_H_2_ was added to the chambers at a headspace concentration of 4% to inhibit CH_4_ oxidation. The chambers were incubated overnight to allow the translocation of the C_2_H_2_ through the plants into the rhizosphere, which would inhibit nearly all microbial CH_4_ oxidation[30,31]. The chambers were removed from the plants for 5 min the next morning to re-establish the ambient atmospheric conditions. The chambers were then redeployed, and the headspace gas was sampled for determining the rate of CH_4_ production as described above for measuring total emission. The rate of CH_4_ oxidation was estimated by subtracting the total CH_4_ emission rate from the CH_4_ production rate.

### Measurement (in situ) of CH_4_ transport in the paddy field

The CH_4_ transport pathways were measured once every two weeks following the method of Wang and Shangguan[32]. The rate of plant-mediated transport was determined by covering the entire surface inside the collar with plastic sheeting and then taping the sheeting to the base of the rice plants to block CH_4_ ebullition and diffusional transport. The rates of total plant-mediated and diffusional transport were determined by covering the surface inside the collar with 0.15-mm gauze and then taping the gauze to the base of the rice plants to block CH_4_ ebullition. The rate of diffusional CH_4_ transport was then determined by subtracting the plant-mediated transport rate from the total plant-mediated and diffusional-transport rates. The rate of CH_4_ ebullition was calculated by subtracting the plant-mediated and diffusional CH_4_ transport rates from the total CH_4_ emission rate. To measure the diurnal variation of GHG emissions we chose the 36 DAT as representing the tiller period and the 85 DAT representing the ripening stage.

### Sampling of porewater and soil samples

Porewater was sampled in situ once every two weeks from April to July 2011. Three specially designed stainless steel tubes (2.0 cm inner diameter) were installed to a depth of 30 cm in each plot. Porewater samples were collected immediately after the measurements of CH_4_ emission using 50-ml syringes, injected into pre-evacuated vials (20 ml), and stored in a cooling box in the field, and another part was injected into the 100 ml sample bottle. After transporting to the laboratory, the samples in the vials were stored at -20°C until the analysis. Three soil porewater samples were randomly collected from the 0–30 cm layer of each plot. The soil samples were collected with a soil sampler (length 0.3 m and diameter 0.1 m) taking a core from the first 15 cm of soil profile.

### Measurement (in situ) of porewater and soil properties

Before analysis, the vials were first thawed at room temperature and were then vigorously shaken for 5 min to equilibrate the CH_4_ concentrations between the porewater and the headspace. The gas samples were taken from the headspace of the vials and analyzed for CH_4_ concentration with the above gas chromatograph[30]. The porewater concentrations of sulfate and dissolved organic carbon (DOC) were determined using a sequence flow analyzer (San^++^, SKALAR Corporation production, Breda, The Netherlands) and a TOC Analyzer (TOC-V CPH, Shimadzu Corporation, Kyoto, Japan), respectively. Fresh soil (0–15 cm) was digested with 1M HCl to determine the total Fe concentration in the soil, and soil Fe^2+^ and Fe^3+^ concentrations were determined using the 1,10-phenanthroline and spectrometric method (UV-2450, Shimadzu Corporation, Kyoto, Japan)[33]. The concentration of soil available N (0–15 cm) was determined by alkaline hydrolysis diffusion [33]. The growth characteristics of rice (e.g. leaf, stem, grain, above- and belowground, and total biomasses) were determined by harvesting three plants (one per plot) on each measurement day, and the biomasses were determined by oven-drying the samples.

### Calculation of CH_4_ and N_2_O fluxes and porewater dissolved CH_4_ concentration

The rates of CH_4_ and N_2_O flux from the paddy field were expressed as the increase/decrease in CH_4_ and N_2_O mass per unit surface area per unit time. CH_4_ and N_2_O fluxes were calculated by:

$$F=\frac{M}{V}\cdot \frac{dc}{dt}\cdot H\cdot (\frac{273}{273+T})$$

where *F* is the CH_4_ or N_2_O flux (mg CH_4_ m^-2^ h^-1^ or μg N_2_O m^-2^ h^-1^), *M* is the molar mass of the respective gas (16 for CH_4_ and 44 for N_2_O), *V* is the molar volume of air at a standard state (22.4 l mol^-1^), *dc/dt* is the change in headspace CH_4_ and N_2_O concentration with time (μmol mol h^-1^), *H* is the height of the chamber above the water surface (m), and *T* is the air temperature inside the chamber (°C).

The concentration of CH_4_ dissolved in the porewater was calculated by Ding et al.[34]:

$$C=\frac{Ch\cdot Vh}{22.4\cdot Vp}$$

where *Ch* is the CH_4_ concentration (μl l^-1^) in the air sample from the vials, *Vh* is the volume of air in the bottle (ml), and *Vp* is the volume of the porewater in the bottle (ml).

### Measurement of environmental and biotic parameters

Ambient air temperature (°C) and air humidity (%) and soil electrical conductivity (mS cm^-1^), Eh (mV), pH, and temperature in the 0–15 cm layer were measured *in situ* each sampling day. Each final measurement was the average of three consecutive measurements. Air temperature and air humidity were measured using a Kestrel 3500 pocket weather meter (N K Scientific Instruments, Carlsbad, USA). Soil redox potential (Eh), pH, and temperature were measured with an Eh/pH/temperature meter (IQ Scientific Instruments, Carlsbad, USA), and soil electrical conductivity was measured using a 2265FS EC Meter (Spectrum Technologies Inc., Paxinos, USA).

### Statistical analysis

The data were checked for normality and homogeneity of variance, and if necessary, were log-transformed. Pearson correlation analysis was also used to determine the relationships of porewater CH_4_ concentration, CH_4_ production, CH_4_ oxidation, CH_4_ transport, CH_4_ emissions, and N_2_O emissions among them and with soil properties, rice growth, and meteorological variables. Stepwise regression analysis was used to determine the relationships of CH_4_ and N_2_O dynamics with soil properties, rice growth, and meteorological variables. All statistical analyses were performed using SPSS Statistics 17.0 (SPSS Inc., Chicago, USA).

## Results

### CH_4_ emissions from the paddy field

**Fig 2:**
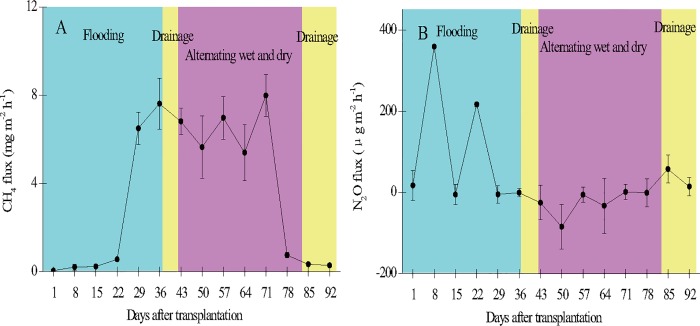
**Seasonal variation of CH**_**4**_ **(A) and N**_**2**_**O (B) fluxes in the paddy field.** Error bars indicate the standard error of the mean of triplicate measurements.

The pattern/intensity of CH_4_ emissions from the paddy field varied with the stage of rice growth (Fig 2). The emission rate was relatively low (0.04–0.55 mg m^-2^ h^-1^) during the initial stage (1–22 DAT) and increased as the crop matured. The rate peaked on 71 DAT (7.99 mg m^-2^ h^-1^) and then decreased following drainage and ripening of the rice crop (0.28–0.75 mg m^-2^ h^-1^). The seasonal average CH_4_ emission rate was 3.53 ± 3.37 mg m^-2^ h^-1^.

**Fig 3:**
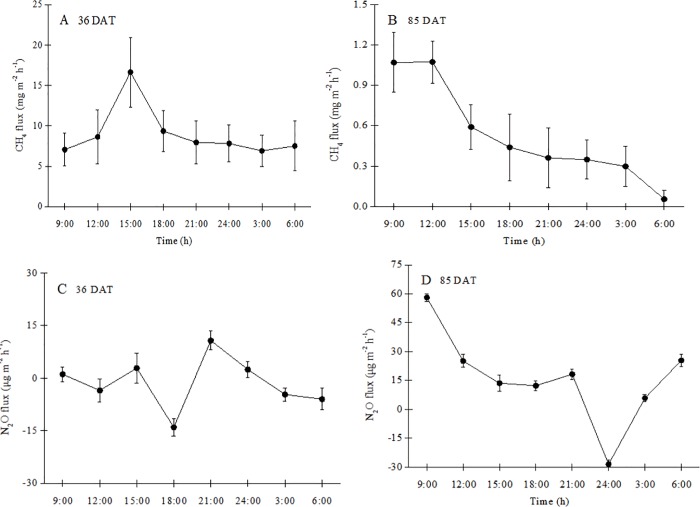
**Diurnal variation of CH**_**4**_ **fluxes in the paddy field 36 (A) and 85 (B) days after transplanting (DAT) and N**_**2**_**O fluxes in the paddy field 36 (C) and 85 (D) DAT.** Error bars indicate the standard error of the mean of triplicate measurements.

Diurnal variation of CH_4_ emissions from the paddy field differed significantly between the tillering (36 DAT) and maturity (85 DAT) stages (Fig 3). On 36 DAT, emission was maximum at 15:00 (9.36 mg m^-2^ h^-1^) and minimum at 9:00 (7.06 mg m^-2^ h^-1^), with an average of 8.98 ± 3.20 mg m^-2^ h^-1^. CH_4_ emissions on 85 DAT decreased continuously throughout the day from a maximum (1.07 mg m^-2^ h^-1^) at 9:00 to a minimum (0.05 mg m^-2^ h^-1^) at 6:00 the next day, with an average of 0.53 ± 0.37 mg m^-2^ h^-1^.

### N_2_O emissions from the paddy field

The pattern/intensity of N_2_O emissions from the wetland paddy field also varied with the stage of rice growth (Fig 2). The emission rate was relatively high (-5.21 to 359 μg m^-2^ h^-1^) during the initial stage (1–22 DAT), but decreased rapidly following the development of anaerobic conditions in the soil. Emission peaked on 8 (359 μg m^-2^ h^-1^) and 22 (217 μg m^-2^ h^-1^) DAT. The emission rate increased slightly after drainage at the final growth stage (-1.00 to 57.4 μg m^-2^ h^-1^). The seasonal average N_2_O emission rate was 36.1 ± 114 μg m^-2^ h^-1^.

Diurnal variation of N_2_O emissions from the wetland paddy field also differed significantly between the tillering (36 DAT) and ripening (85 DAT) stages (Fig 3). On 36 DAT, emission was maximum at 9:00 (10.7 μg m^-2^ h^-1^) and minimum at 6:00 (-14.0 μg m^-2^ h^-1^), with an average of -1.37 ± 7.4 μg m^-2^ h^-1^. On 85 DAT, emission was maximum at 9:00 (58.1 μg m^-2^ h^-1^) and minimum at midnight (-28.5 μg m^-2^ h^-1^), with an average of 16.3 ± 24.1 μg m^-2^ h^-1^.

### Changes in soil, porewater, weather, and plant parameters during the experimental period

**Fig 4:**
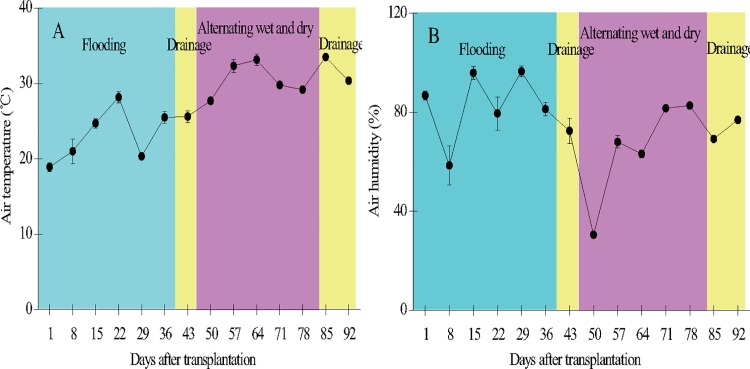
**Temporal variation of air temperature (A) and humidity (B) in the paddy field.** Error bars indicate the standard error of the mean of triplicate measurements.

Ambient air temperature increased steadily over the period of rice growth from 18.9 to 33.5°C, with an average of 27.2 ± 4.7°C. Air humidity varied considerably between 30.6 and 95.9% during the same period, with an average of 74.5 ± 16.8% (Fig 4).

**Fig 5:**
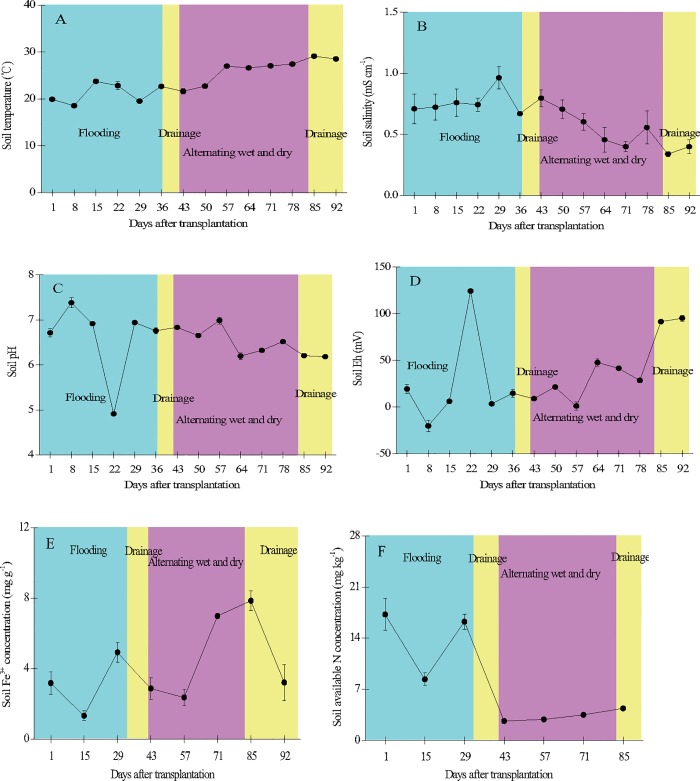
Temporal variation of soil temperature (A), soil salinity (B), pH (C), Eh (D), and Fe^3+^ (E) and available N (F) concentrations in the paddy field. Error bars indicate the standard error of the mean of triplicate measurements.

**Fig 6:**
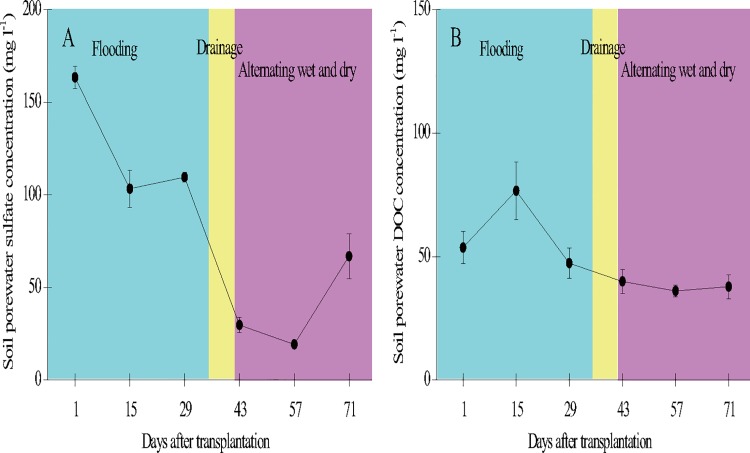
**Temporal variation of soil porewater sulfate (A) and dissolved organic carbon (B) concentrations in the paddy field.** Error bars indicate the standard error of the mean of triplicate measurements.

Various soil parameters (0–15 cm) also changed during the period of growth (Fig 5). Soil temperature increased from 18.5°C at the beginning (April) to 29.1°C at the end (July) of the period, with an average of 24.1 ± 3.5°C. Soil electrical conductivity increased initially and then decreased from 29 DAT onward (range: 0.34–0.96 mS cm^-1^, mean: 0.63 ± 0.18 mS cm^-1^). Soil pH changed significantly throughout the period (range: 4.91–6.99, mean: 6.54 ± 0.58). Soil Eh was lower during the flooding period and began to increase after 64 DAT (range: -20.2 to 124 mV, mean: 34.6 ± 41.7 mV). Soil Fe^3+^ concentration changed with flooding and drainage during the growth period (range: 1.33–7.85 mg g^-1^, mean: 4.21 ± 2.45 mg g^-1^). Soil available N concentration was higher before 29 DAT and began to decrease significantly after 43 DAT (range: 2.66–17.2 mg kg^-1^, mean: 7.90 ± 6.34 mg kg^-1^). Soil porewater sulfate concentration was higher early and late in the growth period and lower during the tillering and flowering stages (range: 19.2–163 mg kg^-1^, mean: 82.0 ± 49.6 mg kg^-1^). Soil porewater DOC concentration increased to a peak on 15 DAT before decreasing (range: 65.6–428 mg kg^-1^, mean: 210 ± 151 mg kg^-1^) (Fig 6).

**Fig 7:**
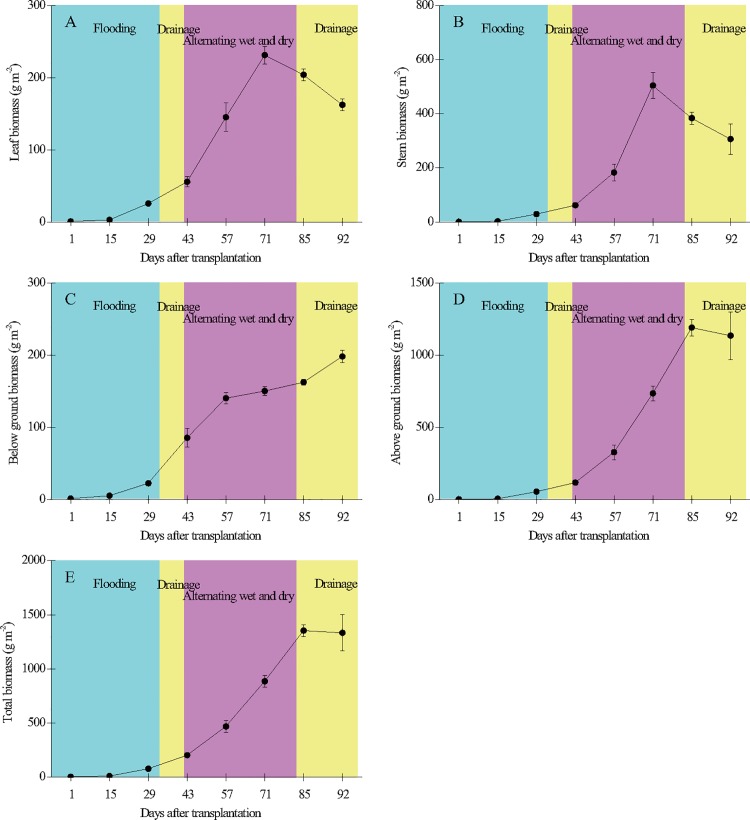
**Temporal variation of plant leaf biomass (A), stem biomass (B), belowground biomass (C), aboveground biomass (D, sum of leaf, stem, and grain biomasses), and total biomass (E, sum of above- and belowground biomasses) in the paddy field.** Error bars indicate the standard error of the mean of triplicate measurements.

The above- and belowground biomasses of the rice plants at harvest were 1135 ± 131 and 198 ± 18.4 g m^-2^, respectively (Fig 7).

### Relationships of CH_4_ production, oxidation, and transport with emissions

**Fig 8:**
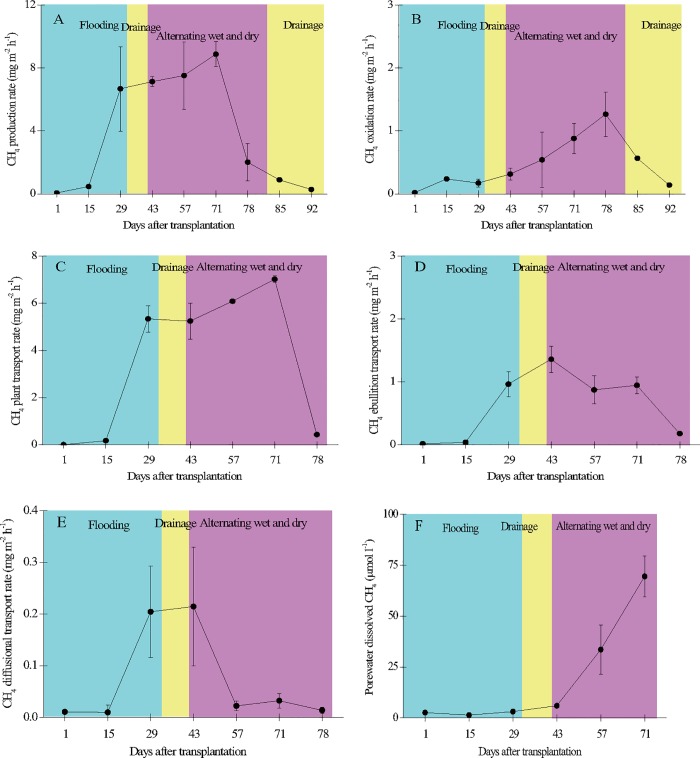
**Seasonal variation of CH**_**4**_ **production (A), oxidation (B), plant transport (C), ebullition transport (D), diffusional transport (E), and porewater dissolved CH**_**4**_ **concentration (F) in the paddy field.** Error bars indicate the standard error of the mean of triplicate measurements.

The rate of CH_4_ production was low early in the season (Fig 8) but had become significantly higher by 71 DAT. The rate of CH_4_ oxidation was low throughout the growth period except during 71–78 DAT. The rate of plant-mediated transport of CH_4_ was low during the initial growth and ripening stages but then rose to a peak on 71 DAT (7.02 mg m^-2^ h^-1^). The rate of CH_4_ ebullition was also low during the initial growth and ripening stages but had increased by 43 DAT (1.36 mg m^-2^ h^-1^). The rate of CH_4_ diffusional transport was low throughout the growth period. Compared to other sampling days, porewater CH_4_ concentrations were significantly higher to a depth of 30 cm on 71 DAT (69.52 μmol l^-1^) and significantly lower on 15 DAT (1.40 μmol l^-1^), with an average of 17.4 μmol l^-1^.

CH_4_ emission was positively correlated with CH_4_ production (*R* = 0.994), plant-mediated transport (*R* = 0.997), ebullition (*R* = 0.940), CH_4_ diffusion (*R* = 0.530), and dissolved CH_4_ concentration in the porewater (*R* = 0.620) (Table 1). About 94% of the CH_4_ produced (calculated as the percentage of production to total emission) from the anaerobic soil environment was released into the atmosphere, and less than 6% was oxidized (Fig 8). Plant-mediated transport was the most important pathway, contributing about 98% of the CH_4_ emission, and ebullition and diffusion together contributed only 2% of the CH_4_ emission.

**Table 1:** Pearson correlation coefficients between CH_4_ emission, production, oxidation, and transport and the concentration of dissolved CH_4._

	CH_4_ emission	CH_4_ production	CH_4_ oxidation	CH_4_ plant transport	CH_4_ ebullition transport	CH_4_ diffusion transport
CH_4_ production (*n* = 27)	0.994**					
CH_4_ oxidation (*n* = 27)	NS	NS				
CH_4_ plant transport (*n* = 21)	0.997**	0.991**	NS			
CH_4_ ebullition transport (*n* = 21)	0.940**	0.924**	NS	0.909**		
CH_4_ diffusional transport (*n* = 21)	0.530**	0.480*	NS	0.467*	0.740**	
Dissolved CH_4_ (*n* = 18)	0.620**	0.661**	0.432*	0.670**	0.378*	NS

*, *P* < 0.05

**, *P* < 0.01

NS, not significant.

### Relationships of CH_4_ metabolism with environmental variables

CH_4_ emission was significantly correlated positively with soil DOC concentration (*R* = 0.667) and some plant biomasses (Table 2) and negatively with soil Eh (*R* = -0.350) and sulfate (*R* = -0.713) and available N concentrations (*R* = -0.370). CH_4_ production was correlated positively with soil DOC concentration (*R* = 0.670) and with some plant biomasses, including total biomass (*R* = 0.562), and negatively with sulfate (*R* = -0.729) and available N (*R* = -0.414) concentrations. CH_4_ oxidation was correlated positively with air temperature (*R* = 0.832) and soil temperature (*R* = 0.838), soil Eh (*R* = 0.480), Fe^3+^ (*R* = 0.582) and DOC concentrations (*R* = 0.368), and all plant-related parameters, including total biomass (*R* = 0.882), and negatively with relative humidity (*R* = -0.520) and soil salinity (*R* = -0.782), pH (*R* = -0.603), and sulfate (*R* = -0.568) and available N concentrations (*R* = -0.771).

**Table 2:** Pearson correlation coefficients between CH_4_ metabolism, N_2_O emission, and various environmental factors.

	CH_4_ emission *n* = 21	CH_4_ production *n* = 21	CH_4_ oxidation *n* = 21	CH_4_ plant transport *n* = 18	CH_4_ ebullition transport *n* = 18	CH_4_ diffusion transport *n* = 18	DissolvedCH_4_ *n* = 18	N_2_O emission *n* = 21
Meteorological factor:								
Temperature	NS	NS	0.832**	0.621**	0.416*	NS	0.728**	NS
Humidity	NS	NS	-0.520**	-0.522*	-0.534*	NS	-0.405*	NS
Soil factor:								
Temperature	NS	NS	0.838**	0.457*	NS	-0.538**	0.810**	0.494*
Salinity	NS	NS	-0.782*	NS	NS	0.649**	-0.889**	-0.651**
pH	NS	NS	-0.603**	NS	NS	NS	-0.730**	-0.728**
Eh	-0.350*	NS	0.480*	NS	NS	NS	0.718**	0.877**
Fe^3+^	NS	NS	0.582**	0.603**	0.413*	NS	0.677**	0.649**
Available nitrogen	-0.370*	-0.414*	-0.771**	-0.560**	-0.532*	NS	-0.565**	NS
Porewater factor:								
Sulfate	-0.713**	-0.729**	-0.568**	-0.732**	-0.746**	NS	-0.422*	0.465*
DOC	0.667**	0.670**	0.368*	0.645**	0.744**	NS	NS	-0.479*
Plant factor:								
Belowground biomass	0.446*	0.480*	0.675**	0.894**	0.936**	0.456*	0.499*	NS
Stem biomass	NS	NS	0.936**	0.673**	0.379*	NS	0.990**	0.445*
Leaf biomass	0.517*	0.563**	0.856**	0.851**	0.690**	NS	0.768**	NS
Aboveground biomass	NS	0.354*	0.939**	0.766**	0.490*	NS	0.977*	0.447*
Total biomass	0.515*	0.562**	0.882**	0.877**	0.706**	NS	0.796*	NS

*, *P* < 0.05

**, *P* < 0.01

NS, not significant.

Plant-mediated transport was correlated positively with air temperature (*R* = 0.621) and soil temperature (*R* = 0.457), soil Fe^3+^ (*R* = 0.603) and DOC concentrations (*R* = 0.645), and all plant-related parameters, including total biomass (*R* = 0.877), and negatively with air humidity (*R* = -0.522) and sulfate (*R* = -0.732) and available N concentrations (*R* = -0.560) (Table 2). Ebullition was correlated positively with air temperature(*R* = 0.416), Fe^3+^ (*R* = 0.413) and DOC (*R* = 0.744) concentrations, and all plant-related parameters, including total biomass (*R* = 0.706), and negatively with relative humidity (*R* = -0.534) and sulfate (*R* = -0.746) and available N concentrations (*R* = -0.532). Diffusional transport was correlated positively with soil salinity (*R* = 0.649) and belowground biomass (*R* = 0.456) and negatively with soil temperature (*R* = -0.538). Dissolved CH_4_ concentration was correlated positively with air temperature (*R* = 0.728) and soil temperatures (*R* = 0.810), Eh (*R* = 0.718), Fe^3+^ concentration (*R* = 0.677), and all plant-related parameters, including total biomass (*R* = 0.796), and negatively with relative humidity (*R* = -0.405) and soil salinity(*R* = -0.889), pH (*R* = -0.730), and sulfate (*R* = -0.422) and available N concentrations (*R* = -0.565).

We used stepwise regression analysis to identify the most important variable(s) controlling CH_4_ metabolism. CH_4_ emission was largely governed by sulfate, available N, and DOC concentrations, stem biomass, air temperature, and relative humidity, which together explained 92% of the variance (Table 3). Sulfate and available N concentrations, stem and leaf biomasses, air temperature, and relative humidity together explained 96% of the variance in CH_4_ production. In contrast, about 88% of the variance in CH_4_ oxidation could be accounted for by changes in aboveground and stem biomasses, air temperature, relative humidity, and soil Eh and available N concentration. Plant-mediated transport of CH_4_ was controlled by above- and belowground biomasses, air temperature, and soil Fe^3+^ concentration and pH, which together explained 91% of the variance. Stem biomass, air and soil temperatures, relative humidity, and soil available N concentration accounted for 69% of the variation in CH_4_ ebullition. Soil salinity and Eh, belowground and leaf biomasses, and air humidity together explained 73% of the variance in CH_4_ diffusional transport. Over 82% of the variance of the dissolved CH_4_ concentration was explained by a combination of stem biomass, relative humidity, and soil temperature, salinity, and available N concentration.

**Table 3:** Equations of stepwise regression analysis for CH_4_ metabolism and N_2_O emission with environmental factors.

Parameter	Equation	*R*^2^
CH_4_ emission	Y = 23.123–0.139 Sulfate + 0.571 available N + 0.017 stem biomass—0.521 air temperature—0.003 DOC—0.003 air humidity	0.92
CH_4_ production	Y = 19.494–0.135 Sulfate + 0.578 available N + 0.018 stem biomass—0.470 air temperature + 0.017 air humidity—0.002 leaf biomass	0.96
CH_4_ oxidation	Y = -0.147 + 0.001 aboveground biomass—0.003 Eh + 0.004 humidity—0.013 available N + 0.001 stem biomass + 0.004 air temperature	0.88
Plant transport	Y = -47.666 + 0.023 belowground biomass + 0.936 Fe^3+^ + 6.690 pH + 0.003 above biomass—0.013 air temperature	0.91
Ebullition transport	Y = -0.696 + 0.011 belowground biomass—0.010 air temperature + 0.009 air humidity + 0.020 available N—0.012 soil temperature	0.69
Diffusional transport	Y = -0.516 + 0.671 soil salinity + 0.001 belowground biomass + 0.004 Eh + 0.001 humidity—0.001 leaf biomass	0.73
Dissolved CH_4_	Y = -36.629 + 0.125 stem biomass + 2.251 soil temperature + 0.872 available N—0.286 air humidity + 5.831 soil salinity	0.82
N_2_O emission	Y = -100.673 + 0.943 Eh + 4.171 available N + 2.697 air temperature—5.614 Fe^3+^ + 0.061 leaf biomass—0.841 pH	0.95

### Relationships of N_2_O emission with environmental variables

Table 2 presents the Pearson correlation coefficients between N_2_O emissions and the environmental factors. N_2_O emission was significantly correlated positively with soil temperature (*R* = 0.494), Eh (*R* = 0.877), Fe^3+^ (*R* = 0.649) and sulfate concentrations (*R* = 0.465), and stem (*R* = 0.445) and aboveground (*R* = 0.447) biomasses and negatively with soil salinity (*R* = -0.651), pH (*R* = -0.728), and DOC concentration (*R* = -0.479) (*P* < 0.05).

The stepwise regression analysis indicated that 95% of the variation in N_2_O emission could be explained by changes in air temperature, leaf biomass, soil Eh, pH, and available N and Fe^3+^ concentrations (Table 3).

### Crop yield and GHG per Mg of crop yield

The crop yield in this paddy field was 8.1 Mg ha^-1^, so CH_4_ and N_2_O emissions per Mg of crop (grain) yield were 9.62 and 0.1 kg, respectively.

## Discussion

### Temporal patterns of CH_4_ and N_2_O emissions

The diurnal variation of CH_4_ emissions from the paddy field differed significantly between the tillering (36 DAT) and ripening (85 DAT) stages of rice growth (Fig 2). Diurnal emission was maximum during the tillering stage at 15:00, which might be attributed to a higher soil temperature in the afternoon that enhanced microbial CH_4_ production. Luo et al.[14] reported that soil temperature had a significant effect on the diurnal variation of CH_4_ emission in a temperate spruce forest in Germany, a tropical rain forest in Australia, and an ungrazed semi-arid steppe in China. The diurnal variation of CH_4_ emission may also be partly explained by the photosynthetic activity of the rice plants. Up to 52% of the CH_4_ emissions from paddy soils comes the exudation of labile organic carbon from roots to the rhizosphere for methanogenesis i.e. comes from photosynthesis. The other 48% is emitted from old soil carbon[35]. The photosynthetic activity of rice plants during the day probably causes a rapid translocation of photosynthates to belowground tissues, hence promoting CH_4_ production and emissions from soils[35, 36]. Lai et al.[36] reported that the duration of CH_4_ production was much shorter than the lag in CH_4_ flux of 9–12 hours after maximum photosynthetic activity in a sedge-dominated community in a northern peatland. Microbial respiration in soils may also increase CO_2_ concentrations in soil water due to the absence of photosynthesis at night, because the CO_2_ cannot be used for photosynthesis. Higher CO_2_ concentrations could reduce the pH of soil solutions, which in turn could lower CH_4_ production and emissions from soils at night. The diurnal CH_4_ emission during ripening was maximum at 9:00. Young rice plants contributed a substantial proportion of their photosynthates to soils compared to mature plants[37]. The diurnal variability of CH_4_ flux during ripening was likely not strongly limited by the supply of labile carbon substrates, but by other environmental factors. The CH_4_ peak in the early morning could be attributed to wind-driven ventilation immediately after sunrise that promoted the mass transport of CH_4_ produced and stored in soil pores during the relatively calm night.

CH_4_ emission, production, and plant-mediated transport had similar seasonal patterns, with higher rates on 71 DAT (young panicle differentiation stage) and lower rates during the initial growth and ripening stages (Fig 8). Such seasonal patterns were typical for CH_4_ metabolism in paddy fields, especially for the overall CH_4_ emission, with similar findings reported in Italy[38], Japan[39], and northeastern China[40].

The diurnal variation of N_2_O emissions from the paddy field differed significantly between the tillering (36 DAT) and ripening (85 DAT) stages (Fig 2). Emissions during the tillering stage were maximum and minimum at 9:00 and 18:00, respectively, similar to emissions in a natural wetland [41]. Emission during ripening was maximum at 9:00. A combination of low nighttime temperature and N_2_O diffusion rate from the paddy soil to the atmosphere likely led to the accumulation of N_2_O in the soil. The accumulated N_2_O would be released to the atmosphere the following morning in a burst due to turbulence-driven mass flow. Higher daytime N_2_O emission has also been reported for mangroves during the early growth stage [20, 42], which could be due to changes in N chemistry, O_2_ availability, or soil temperature[43, 44].

### Main CH_4_ metabolic process controlling CH_4_ emission

Frenzel and Karofeld[10] reported that 80–99% of the CH_4_ produced in paddy soil was oxidized in the rhizosphere, and Jia et al.[45] reported that an average of 36.3–54.7% of the CH_4_ produced was oxidized in rice paddy soils. The fraction of the CH_4_ oxidized in our study was much smaller (9.6%), perhaps due to a smaller methanotrophic community, less efficient transport of oxygen to the rhizosphere, or both. Further studies of microbial diversity and anaerobic CH_4_ oxidation are required to elucidate the causes of the limited role of methanotrophs in the paddy soil.

Plant-mediated transport was the most important pathway of CH_4_ transport (about 98% of total CH_4_ emission). Ebullition and diffusion contributed only 2% to the overall CH_4_ emission. Frenzel and Karofeld[10] reported that ebullition accounted for only <1% of the total CH_4_ emitted to the atmosphere from a raised bog, which was similar in magnitude to our findings. Boose and Frenzel[46] found a strong influence of ebullition on CH_4_ emissions from rice microcosms during the early vegetative and late senescence phases, but such variations among growth stages were not seen in the present study. The high CH_4_ emissions at the young panicle differentiation stage (71 DAT) might be attributed to the rapid development of plant aerenchyma, which is supported by the higher rate of plant-mediated CH_4_ transport on 71 DAT (Fig 6). Jia et al.[45], however, reported a higher CH_4_ emission from paddy soils during the tillering stage compared to the panicle initiation stage, which could be explained by less oxidation of the produced CH_4_. CH_4_ oxidation at our study site, though, did not have a significant influence on overall CH_4_ emissions. The relationships among CH_4_ emissions production and transport, but not with CH_4_ oxidation, suggest that CH_4_ emissions are mainly related with CH_4_ production and CH_4_ transport and less or not with CH4 oxidation.

### Influence of environmental factors on CH_4_ and N_2_O emission: clues for mitigation

Both CH_4_ production and emission in this subtropical paddy field were predominantly controlled by the soil concentrations of sulfate and DOC and the biomass of the rice plants. Sulfate concentration was the most important factor controlling CH_4_ emission due to the inhibitory effects of sulfate on the activities of soil methanogens during rice cultivation[47]. Our study demonstrated significant and negative correlations between soil sulfate concentration and CH_4_ production and emission, in accordance with the results from other studies simulating the effects of sulfate deposition on paddy fields [47–49]. Sulfate reduction is thermodynamically more favorable than CH_4_ production in the anaerobic degradation of soil organic matter[50], so an increase in the availability of sulfate ions would suppress the activity of methanogens and CH_4_ production during rice cultivation[51]. Acetate and molecular hydrogen (H_2_) are important methanogenic substrates but may also be consumed by oxidation with electron acceptors such as sulfate[50]. An increase in soil sulfate concentration in an Italian paddy field reduced the H_2_ partial pressure in the soil below the threshold concentration for methanogens, thus inhibiting H_2_-dependent methanogenesis[51]. The drainage of paddy fields in Texas, USA, reduced acetate concentrations and concomitantly increased the soil sulfate concentration and decreased CH_4_ production, suggesting that sulfate reducers were able to outcompete methanogens for the available acetate, a labile carbon substrate [51]. But we must be careful with the direct link between sulfate concentration and CH_4_ release because of the max value of CH_4_ emission occurred during flood period, and at the as time, sulfate reduction is also very high due to the redox state in anaerobic environment. Therefore, this relationship might be driven by the water condition in the paddy field, and does not mean a causal relationship.

Sulfate concentration in our study, however, was significantly and positively correlated with soil N_2_O emissions and was governed by mechanisms similar to the effects of Fe^3+^ on N_2_O emission. Sulfate, as an oxidant, could facilitate the oxidation of NH_4_^+^, which could then enhance the production of N_2_O[52]. Moreover, an increase in sulfate concentration would increase the amount of elemental sulfur in the paddy field, which could act as a reductant and enhance the production of N_2_O by reducing NO_3_^-^[53].

Many agricultural management practices have been developed for mitigating CH_4_ and N_2_O emissions from paddy fields[54–56]. Ali et al.[57] reported that intermittent irrigation of rice paddies significantly decreased CH_4_ emission but stimulated N_2_O emission. Kudo et al.[56] also reported that mid-season drainage successfully mitigated CH_4_ emissions from paddy fields but led to a sharp increase in N_2_O emissions. The results from these studies suggest a trade-off between mitigating CH_4_ and N_2_O fluxes from paddy fields. N_2_O is a more potent GHG than CH_4_, especially on a long-term basis, so the overall global-warming potential of the various management strategies should be carefully considered[58].

Our study showed that CH_4_ emission from a subtropical paddy field was negatively correlated with N_2_O emission, which may mainly have been due to the differential responses of various microbial groups (e.g. methanogens and nitrifying and denitrifying bacteria) to critical environmental factors[59]. For example, sufficiently low Eh is required for CH_4_ formation, because methanogenic bacteria metabolize organic substances under strictly anaerobic conditions[60]. Soil denitrification, however, could become dominant under such conditions, and most of the intermediate products (i.e. NO and N_2_O) could be reduced to the final product N_2_, especially in N-limited areas such as our study site[61] where N_2_O emission can be negative (Fig 2). Van Rijn[62], however, reported that denitrification in soils with a high Eh could become a dominant microbial process that promotes N_2_O production but inhibits methanogenic activity.

DOC concentration is an indicator of the availability of substrates for CH_4_ production that in turn influences CH_4_ emission[63]. A large amount of DOC could be derived from the exudation of organic acids from roots, and about 61–83% of the carbon in these exudates could serve as methanogenic substrates and eventually be converted into CH_4_[36]. The concentration of soil organic carbon was lower at our study site (18.1 g kg^−1^) than in other paddy fields[64, 65]. CH_4_ production in our paddy field was thus probably limited by the availability of organic substrates, which was supported by the positive response of CH_4_ production to carbon addition reported for wetland soils in the same study area[66]. The observed increasing concentration of methane dissolved in pore water after 43 DAT (Fig 8) coincided with the re-flooded period after the drainage period when much carbon from roots and plants has been deposited in the soil, and therefore was available in pore water.

The rice plants were an important factor controlling CH_4_ emissions from the soil. The development of an internal gas-space ventilation system (aerenchyma) in some vascular plants provides improved aeration for submerged organs in anoxic soils below the water table[67]. These aerenchymatous tissues can also serve as gas conduits for the transport of CH_4_ from the rhizosphere to the atmosphere, while bypassing the aerobic, methane-oxidizing layers[68]. Molecular diffusion and bulk flow are two other mechanisms involved in the transport of CH_4_ from soil to the atmosphere[67]. The respiratory uptake of oxygen by plants creates a diffusion gradient that draws O_2_ from the atmosphere to the rhizosphere[17]. This gradient is accompanied by an upward diffusion of CH_4_ from the rhizosphere to the atmosphere via the aerenchyma down the concentration gradient. The bulk transport of CH_4_ by plants, though, is driven by pressure differences. Differences in temperature or water-vapor pressure between the internal air spaces in plants and the surrounding atmosphere generate a pressure gradient that drives gases to vent from leaves to the rhizosphere in bulk and then vent back to the atmosphere through old leaves or rhizomes connected to other shoots. During this convective flow, CH_4_ produced in the rhizosphere can also be rapidly flushed to the atmosphere[67]. This plant-mediated pathway enables an efficient transport of CH_4_ with minimal resistance[17].

The N_2_O emissions from the paddy field varied with the stage of rice growth. Emissions were higher during initial growth compared to other stages due to the increased availability of substrates for N_2_O production subsequent to the N fertilization, in agreement with previous studies[69]. Our stepwise regression analysis also indicated that the fluctuation of soil Eh between the alternating wet and dry periods was an important factor influencing N_2_O emission, consistent with the findings of similar studies[70]. Furthermore, the soil Fe^3+^ concentration was significantly and positively correlated with N_2_O emission (Table 2). Previous studies have also found that variations in N_2_O emissions from subtropical paddy fields were dependent on Fe^3+^ concentrations during rice growth, which may be due to two mechanisms[71, 72]. Firstly, higher Fe^3+^ concentrations could increase the biological oxidation of ammonium that is known to generate N_2_O by nitrification[73]. Secondly, higher concentrations of Fe^3+^ in the soil could also lead to an increase in Fe^2+^ concentrations, which in turn would promote the reduction of nitrite to N_2_O [54, 73].

The results of our study also indicated a significant, positive correlation between soil Eh and N_2_O emission. A decrease in Eh in a subtropical swamp was accompanied by a reduction in soil N_2_O concentrations that could directly reduce the emissions of N_2_O to the atmosphere[74]. Previous studies have also reported higher emissions of N_2_O during the day than at night as a result of higher dissolved O_2_ concentrations, which indirectly supported the findings of a positive correlation between soil Eh and N_2_O emission[43, 44]. Based on our results and those from previous studies, the positive relationship between N_2_O emission and Eh in our paddy field was likely because nitrification was the major mechanism governing N_2_O production, as supported by the very low concentrations of dissolved NO_3_^-^ measured in the porewater samples that were below the detection limit of our ion chromatograph (<0.01 mg l^-1^, ICS2100, Dionex Corporation, Sunnyvale, USA).

This study provides results indicating that both models of CH_4_ emissions and management strategies to reduce CH_4_ emissions should take into account the trade-off between N_2_O and CH_4_ emissions. For instance, some previous studies claim that the use of sulphate N-fertilization reduced CH_4_ emissions[11], however, as commented this could increase N_2_O emissions. The limited number of available observations of CH_4_ N_2_O emissions in relation to environmental variables under field conditions have constrained the parameterization and validation of process-based biogeochemistry models [15, 16]; this study can thus contribute to improve models of CH_4_ and N_2_O emissions. Moreover, the results of this study indicate the interest of management strategies that can reduce methane emission by decreasing the production or transportation or increasing the oxidation. Furthermore, application of electron acceptors, such as Fe^2+^, SO_4_^2-^, or NO_3_^-^ could also be considered.

## Conclusions

Methanogenesis and plant-related CH_4_ transport are the two main processes governing the overall CH_4_ emission from paddy fields. The reduction of CH_4_ production in soil is thus critical for any attempt to mitigate CH_4_ emissions from paddy fields. The sulfate concentrations were negatively correlated with CH_4_ emissions, so the amendment with sulfate fertilizers may be a viable option to reduce CH_4_ emission from the soil. Acid and sulfate deposition (by rainwater) are increasing in this part of China, so CH_4_ production and emissions would likely be suppressed to some extent due to an increase in sulfate availability. Our results, however, suggest that the use of sulfate fertilizer may increase N_2_O emissions from paddy fields. The stepwise regression analysis showed that plant-related parameters, such as stem and leaf biomasses, were the most important factors controlling CH_4_ and N_2_O emissions with significant, positive correlations. Rice cultivars with low biomasses should thus be selected for reducing the plant-mediated transport of CH_4_ and N_2_O through the aerenchymatous tissue. CH_4_ emission was positively correlated with DOC concentration, so our results suggest that the addition of carbon substrates such as straw could be an option for mitigating the GHG emissions from paddy fields.

## Supporting Information

S1 Appendix Field data used in this study. (DOC)
