# Supplementary material for: Factors Related with CH4 and N2O Emissions from a Paddy Field: Clues for Management implications
Source: PLoS One. 2017 Jan 12;12(1):e0169254. doi: 10.1371/journal.pone.0169254 (PMC5230764; doi:10.1371/journal.pone.0169254)
Supplement: S1 Appendix — (DOC) [file pone.0169254.s001.doc]

**Appendix S1**

**Data submitted to Plos one**

| Date | CH_4_ emission (mg m^-2^ h^-1^) | SE |
| --- | --- | --- |
| 1 | 0.04 | 0.01 |
| 8 | 0.21 | 0.14 |
| 15 | 0.23 | 0.01 |
| 22 | 0.56 | 0.03 |
| 29 | 6.51 | 0.73 |
| 36 | 7.62 | 1.16 |
| 43 | 6.82 | 0.60 |
| 50 | 5.65 | 1.43 |
| 57 | 6.98 | 0.97 |
| 64 | 5.40 | 1.28 |
| 71 | 8.00 | 0.96 |
| 78 | 0.75 | 0.12 |
| 85 | 0.33 | 0.04 |
| 92 | 0.28 | 0.08 |
|  |  |  |
| Date | N_2_O emission (μg m^-2^ h^-1^) | SE |
| 1 | 16.93 | 37.02 |
| 8 | 358.83 | 5.89 |
| 15 | -5.21 | 24.38 |
| 22 | 216.66 | 5.75 |
| 29 | -4.90 | 21.49 |
| 36 | -0.73 | 10.18 |
| 43 | -25.14 | 42.16 |
| 50 | -84.77 | 54.44 |
| 57 | -5.49 | 18.96 |
| 64 | -32.88 | 67.69 |
| 71 | 1.29 | 18.65 |
| 78 | -1.00 | 34.06 |
| 85 | 57.36 | 34.52 |
| 92 | 14.34 | 22.73 |
| 36 DAT | CH_4_ diurnal emission (mg m^-2^ h^-1^) | SE |
| 9:00 | 7.06 | 2.05 |
| 12:00 | 8.64 | 3.33 |
| 15:00 | 16.65 | 4.31 |
| 18:00 | 9.36 | 2.51 |
| 21:00 | 7.95 | 2.68 |
| 24:00 | 7.83 | 2.30 |
| 3:00 | 6.91 | 1.91 |
| 6:00 | 7.51 | 3.09 |
| 85 DAT | CH_4_ diurnal emission (mg m^-2^ h^-1^) | SE |
| 9:00 | 1.07 | 0.22 |
| 12:00 | 1.07 | 0.16 |
| 15:00 | 0.59 | 0.17 |
| 18:00 | 0.44 | 0.25 |
| 21:00 | 0.36 | 0.22 |
| 24:00 | 0.35 | 0.14 |
| 3:00 | 0.30 | 0.15 |
| 6:00 | 0.05 | 0.06 |
| 36 DAT | N_2_O diurnal emission (μg m^-2^ h^-1^) | SE |
| 9:00 | 1.11 | 2.05 |
| 12:00 | -3.50 | 3.33 |
| 15:00 | 2.88 | 4.31 |
| 18:00 | -14.02 | 2.51 |
| 21:00 | 10.73 | 2.68 |
| 24:00 | 2.48 | 2.30 |
| 3:00 | -4.63 | 1.91 |
| 6:00 | -5.97 | 3.09 |
| 85 DAT | N_2_O diurnal emission (μg m^-2^ h^-1^) | SE |
| 9:00 | 58.08 | 2.05 |
| 12:00 | 25.12 | 3.33 |
| 15:00 | 13.57 | 4.31 |
| 18:00 | 12.28 | 2.51 |
| 21:00 | 18.27 | 2.68 |
| 24:00 | -28.53 | 2.30 |
| 3:00 | 5.81 | 1.91 |
| 6:00 | 25.42 | 3.09 |
| Date | Air temperature (℃) | SE |
| 1 | 18.90 | 0.61 |
| 8 | 21.00 | 1.65 |
| 15 | 24.70 | 0.61 |
| 22 | 28.17 | 0.76 |
| 29 | 20.33 | 0.03 |
| 36 | 25.50 | 0.76 |
| 43 | 25.60 | 0.79 |
| 50 | 27.70 | 0.21 |
| 57 | 32.33 | 0.87 |
| 64 | 33.13 | 0.74 |
| 71 | 29.80 | 0.35 |
| 78 | 29.17 | 0.17 |
| 85 | 33.50 | 0.21 |
| 92 | 30.37 | 0.27 |
| Date | Air humidity (%) | SE |
| 1 | 86.77 | 1.84 |
| 8 | 58.50 | 7.87 |
| 15 | 95.93 | 2.60 |
| 22 | 79.50 | 6.74 |
| 29 | 96.50 | 2.11 |
| 36 | 81.27 | 2.79 |
| 43 | 72.50 | 5.01 |
| 50 | 30.57 | 0.87 |
| 57 | 68.03 | 2.50 |
| 64 | 63.17 | 1.56 |
| 71 | 81.63 | 0.57 |
| 78 | 82.70 | 1.35 |
| 85 | 69.20 | 0.66 |
| 92 | 76.93 | 0.78 |
| Date | Soil temperature (℃) | SE |
| 1 | 19.93 | 0.15 |
| 8 | 18.53 | 0.13 |
| 15 | 23.77 | 0.09 |
| 22 | 22.83 | 0.84 |
| 29 | 19.53 | 0.18 |
| 36 | 22.67 | 0.27 |
| 43 | 21.63 | 0.55 |
| 50 | 22.73 | 0.09 |
| 57 | 27.00 | 0.17 |
| 64 | 26.63 | 0.15 |
| 71 | 27.07 | 0.03 |
| 78 | 27.43 | 0.03 |
| 85 | 29.13 | 0.30 |
| 92 | 28.53 | 0.41 |
| Date | Soil salinity (mS cm^-1^) | SE |
| 1 | 0.71 | 0.12 |
| 8 | 0.72 | 0.11 |
| 15 | 0.76 | 0.11 |
| 22 | 0.74 | 0.05 |
| 29 | 0.96 | 0.09 |
| 36 | 0.67 | 0.02 |
| 43 | 0.80 | 0.07 |
| 50 | 0.71 | 0.08 |
| 57 | 0.60 | 0.07 |
| 64 | 0.46 | 0.10 |
| 71 | 0.40 | 0.04 |
| 78 | 0.56 | 0.13 |
| 85 | 0.34 | 0.02 |
| 92 | 0.40 | 0.06 |
| Date | Soil pH | SE |
| 1 | 6.71 | 0.09 |
| 8 | 7.38 | 0.11 |
| 15 | 6.92 | 0.03 |
| 22 | 4.91 | 0.01 |
| 29 | 6.94 | 0.02 |
| 36 | 6.76 | 0.07 |
| 43 | 6.83 | 0.04 |
| 50 | 6.65 | 0.01 |
| 57 | 6.99 | 0.08 |
| 64 | 6.20 | 0.07 |
| 71 | 6.32 | 0.05 |
| 78 | 6.52 | 0.04 |
| 85 | 6.21 | 0.01 |
| 92 | 6.18 | 0.02 |
| Date | Soil Eh (mV) | SE |
| 1 | 19.30 | 4.82 |
| 8 | -20.20 | 5.92 |
| 15 | 6.03 | 0.85 |
| 22 | 124.30 | 1.43 |
| 29 | 3.43 | 0.81 |
| 36 | 14.73 | 4.14 |
| 43 | 8.97 | 1.53 |
| 50 | 21.53 | 1.37 |
| 57 | 1.17 | 4.45 |
| 64 | 47.73 | 3.98 |
| 71 | 41.57 | 1.72 |
| 78 | 28.60 | 2.45 |
| 85 | 91.53 | 1.16 |
| 92 | 95.13 | 3.09 |
| Date | Soil Fe^3+^ concentration (mg g^-1^) | SE |
| 1 | 3.18 | 0.63 |
| 15 | 1.33 | 0.27 |
| 29 | 4.92 | 0.55 |
| 43 | 2.87 | 0.62 |
| 57 | 2.36 | 0.47 |
| 71 | 6.99 | 0.12 |
| 85 | 7.85 | 0.56 |
| 92 | 3.21 | 1.02 |
| Date | Soil available N concentration (mg kg^-1^) | SE |
| 1 | 17.23 | 2.17 |
| 15 | 8.37 | 0.88 |
| 29 | 16.25 | 1.01 |
| 43 | 2.66 | 0.06 |
| 57 | 2.88 | 0.20 |
| 71 | 3.50 | 0.15 |
| 85 | 4.39 | 0.24 |
| Date | Soil porewater sulfate concentration (mg l^-1^) | SE |
| 1 | 163.37 | 6.06 |
| 15 | 103.24 | 9.98 |
| 29 | 109.53 | 2.65 |
| 43 | 29.74 | 4.00 |
| 57 | 19.22 | 1.54 |
| 71 | 66.89 | 12.13 |
| Date | Soil porewater DOC concentration (mg l^-1^) | SE |
| 1 | 53.66 | 6.49 |
| 15 | 76.60 | 11.65 |
| 29 | 47.38 | 6.17 |
| 43 | 39.93 | 4.92 |
| 57 | 36.08 | 2.44 |
| 71 | 37.80 | 4.85 |
| Date | Leaf biomass (g m^-2^) | SE |
| 1 | 0.70 | 0.06 |
| 15 | 2.80 | 0.26 |
| 29 | 25.59 | 1.70 |
| 43 | 55.64 | 7.16 |
| 57 | 145.14 | 19.59 |
| 71 | 231.20 | 12.44 |
| 85 | 204.00 | 8.31 |
| 92 | 162.40 | 8.27 |
| Date | Stem biomass (g m^-2^) | SE |
| 1 | 0.56 | 0.02 |
| 15 | 2.22 | 0.07 |
| 29 | 29.08 | 2.56 |
| 43 | 61.74 | 1.51 |
| 57 | 182.56 | 31.38 |
| 71 | 504.40 | 47.56 |
| 85 | 383.60 | 22.41 |
| 92 | 306.40 | 55.95 |
| Date | Below ground biomass (g m^-2^) | SE |
| 1 | 1.32 | 0.09 |
| 15 | 5.30 | 0.35 |
| 29 | 22.50 | 3.39 |
| 43 | 85.54 | 13.12 |
| 57 | 140.33 | 7.86 |
| 71 | 150.40 | 6.11 |
| 85 | 162.53 | 4.15 |
| 92 | 198.21 | 8.27 |
| Date | Above ground biomass (g m^-2^) | SE |
| 1 | 1.26 | 0.05 |
| 15 | 5.02 | 0.21 |
| 29 | 54.66 | 4.21 |
| 43 | 117.38 | 8.65 |
| 57 | 327.70 | 50.93 |
| 71 | 735.60 | 51.20 |
| 85 | 1191.20 | 57.20 |
| 92 | 1135.20 | 164.32 |
| Date | Total ground biomass (g m^-2^) | SE |
| 1 | 2.58 | 0.05 |
| 15 | 10.32 | 0.22 |
| 29 | 77.17 | 7.58 |
| 43 | 202.93 | 17.84 |
| 57 | 468.03 | 54.09 |
| 71 | 886.00 | 53.02 |
| 85 | 1353.73 | 54.04 |
| 92 | 1333.41 | 167.52 |
| Date | CH_4_ production (mg m^-2^ h^-1^) | SE |
| 1 | 0.06 | 0.01 |
| 15 | 0.47 | 0.04 |
| 29 | 6.68 | 2.68 |
| 43 | 7.14 | 0.31 |
| 57 | 7.52 | 2.13 |
| 71 | 8.88 | 0.80 |
| 78 | 2.02 | 1.18 |
| 85 | 0.90 | 0.04 |
| 92 | 0.28 | 0.09 |
| Date | CH_4_ oxidation (mg m^-2^ h^-1^) | SE |
| 1 | 0.02 | 0.00 |
| 15 | 0.24 | 0.01 |
| 29 | 0.17 | 0.07 |
| 43 | 0.32 | 0.09 |
| 57 | 0.54 | 0.44 |
| 71 | 0.88 | 0.24 |
| 78 | 1.27 | 0.35 |
| 85 | 0.57 | 0.01 |
| 92 | 0.14 | 0.03 |
| Date | CH_4_ plant transport (mg m^-2^ h^-1^) | SE |
| 1 | 0.01 | 0.00 |
| 15 | 0.18 | 0.01 |
| 29 | 5.34 | 0.57 |
| 43 | 5.25 | 0.77 |
| 57 | 6.09 | 0.09 |
| 71 | 7.02 | 0.14 |
| 78 | 0.43 | 0.04 |
| Date | CH_4_ ebullition transport (mg m^-2^ h^-1^) | SE |
| 1 | 0.02 | 0.01 |
| 15 | 0.04 | 0.01 |
| 29 | 0.97 | 0.20 |
| 43 | 1.36 | 0.21 |
| 57 | 0.87 | 0.22 |
| 71 | 0.95 | 0.13 |
| 78 | 0.18 | 0.01 |
| Date | CH_4_ diffusional transport (mg m^-2^ h^-1^) | SE |
| 1 | 0.01 | 0.00 |
| 15 | 0.01 | 0.01 |
| 29 | 0.20 | 0.09 |
| 43 | 0.21 | 0.11 |
| 57 | 0.02 | 0.01 |
| 71 | 0.03 | 0.01 |
| 78 | 0.01 | 0.01 |
| Date | Porewater dissolved CH_4_ (μmol l^-1^) | SE |
| 1 | 2.63 | 0.41 |
| 15 | 1.40 | 0.10 |
| 29 | 3.14 | 0.39 |
| 43 | 6.01 | 0.91 |
| 57 | 33.57 | 12.06 |
| 71 | 69.52 | 10.14 |
